# Prise en charge des corps étrangers du tiers supérieur de l’œsophage sans endoscope: un défi pour le médecin en situation isolée?

**DOI:** 10.11604/pamj.2018.30.42.12628

**Published:** 2018-05-18

**Authors:** Antoine Lamblin, Clément Derkenne, Antoine Schwartz, Pierre Pasquier, Romain Gorioux, Pierre-François Wey

**Affiliations:** 1 Service d’Anesthésie-Réanimation, Hôpital d’Instruction des Armées Percy, Clamart, France; 2 Brigade des Sapeurs-Pompiers, Paris, France; 3 Service de Chirurgie Viscérale, Lyon, France; 4 Service de Chirurgie Orthopédique, Hôpital d’Instruction des Armées Desgenettes, Lyon, France; 5 Service d'Anesthésie-Réanimation, Hôpital d'Instruction des Armées Desgenettes, Lyon, France

**Keywords:** Corps étranger, tiers-supérieur de l´œsophage, extraction, Foreign body, upper third of the esophagus, removal

## Abstract

La prise en charge des corps étrangers du tiers supérieur de l'œsophage est bien codifiée et fait appel le plus souvent à un traitement endoscopique. Ce matériel spécifique, ainsi que des médecins gastro-entérologues entraînés à son utilisation fait parfois défaut dans les pays d'Afrique. D'autres moyens doivent alors être envisagés pour les médecins ayant à prendre en charge ce type de pathologie. L'objectif de cette étude était d'évaluer la prise en charge des corps étrangers du tiers supérieur de l'œsophage par des médecins anesthésistes-réanimateurs dans un centre médico-chirurgical du Tchad. 37 cas d'extraction chez des enfants de 0 à 15 ans étaient analysés. Il s'agissait de pièces de monnaie et de piles au lithium dans respectivement 92% et 8% des cas. La méthode employée était une sonde de Foley à ballonnet dans 43,2% et d'une pince à calcul sous laryngoscopie dans 56,8% des cas. Un cas d'échec ayant nécessité une cervicotomie était noté dans chaque groupe. Quatre complications mineures étaient rapportées dans le groupe « pince à calcul », aucune dans le groupe « Foley ». Les indications d'extraction de corps étrangers du tiers supérieur de l'œsophage doivent être bien connues des médecins amenés à les prendre en charge. En cas d'indisponibilité de l'endoscopie, d'autres techniques alternatives peuvent être utilisées avec un taux de succès satisfaisant. En cas d'échec un recours à la chirurgie peut être nécessaire.

## Abstract (en)

The treatment of foreign bodies in the upper third of the esophagus is well codified and it is more often based on endoscopic treatment. This specific instrument as well as gastroenterologists trained in its use are sometimes missing in African countries. Physicians have to use alternative approaches to treat this disease. This study aimed to evaluate the procedure for the removal of foreign bodies in the upper third of the esophagus carried out by anaesthetists-resuscitators in a medical and surgical center in Chad. Thirty seven cases of foreign body removal in children aged 0-15 years were analyzed. Coins and lithium batteries were removed in 92% and 8% of cases respectively. Foley balloon catheter was used in 43.2% of cases and laryngoscopy forceps in 56.8% of cases. One case of therapeutic failure requiring cervicotomy was recorded in each group. Four minor complications were reported in the “laryngoscopy forceps group”, no complication in the “Foley” group. Physicians must have adequate preparation for the removal of a foreign body in the upper third of the esophagus. In case of unavailability of endoscope, other alternative techniques can be used with a satisfying success rate. In case of failure, surgery may be necessary.

## Introduction

L'ingestion de corps étrangers (CE) n'est pas une pathologie rare. Elle intéresse surtout les patients aux deux extrêmes de la vie. Le diagnostic repose sur l'interrogatoire du patient ou de son entourage à la recherche d'un syndrome de pénétration, d'une douleur rétrosternale, d'une hypersialorrhée, d'une dysphagie ou de vomissements. L'examen physique et le bilan radiologique sont systématiques afin d'identifier le site du blocage, le type de CE (dans 80% des cas situés dans l'œsophage), mais également les potentielles complications associées [1]. Les CE de l'hypopharynx et de l'œsophage obstructifs, coupants ou tranchants, les piles plates, ainsi que ceux dont les dimensions dépassant 2,5cm d'épaisseur et 6cm de long doivent être retirés en urgence. L'extraction du CE s'effectue sous anesthésie générale à l'aide d'un laryngoscope (CE situés au-dessus du muscle crico-pharyngien) ou d'un endoscope assorti d'une pince, d'un panier ou d'un ballonnet de type Fogarty (CE situés en dessous du muscle crico-pharyngien). Les suites opératoires sont souvent simples mais nécessitent l'emploi d'anti-inflammatoires stéroïdiens si l'extraction a été traumatique. L'intubation orotrachéale est toujours recommandée [2]. Si l'endoscopie est d'accès aisé dans les grandes métropoles, ce n'est pas forcément le cas dans certains pays d'Afrique Sub-Saharienne où l'accès aux moyens d'endoscopie souple comme rigide fait parfois défaut, tout comme la présence de médecins spécialisés en gastro-entérologie. Dès lors il faut envisager d'autres moyens d'extraction que l'endoscopie pour les CE situés en dessous du muscle crico-pharyngien. Cette étude propose d'analyser les méthodes employées pour l'extraction de 37 cas de CE oesophagiens sous crico-pharyngiens, réalisées dans un centre hospitalier d'Afrique Sub-Saharienne non muni de moyens d'endoscopie ni de spécialiste en gastro-entérologie.

## Méthodes

Il s'agissait d'une étude rétrospective du 01/01/2005 au 01/04/2017 monocentrique, réalisée au pôle de santé unique (PSU) du camp militaire français Adji Kossei de N'Djamena, centre médico-chirurgical doté de 42 lits, réalisant en moyenne 1200 interventions chirurgicales par an dont plus de 90% au profit de la population Tchadienne. L'équipe médicale est composée d'un médecin anesthésiste-réanimateur, d'un chirurgien viscéraliste et d'un chirurgien orthopédiste. Les données étaient recueillies par l'analyse des dossiers informatisés des patients admis pour CE. Les critères d'inclusion étaient: 1/présence d'un CE du tiers supérieur de l'œsophage objectivé par une radiographie (CE radio-opaques uniquement) 2/âge inférieur ou égal à 15 ans. Les critères d'exclusion étaient: 1/âge supérieur à 15 ans 2/corps étranger du tiers moyen ou inférieur de l'œsophage, gastrique ou trachéal. Les données recueillies étaient les caractéristiques démographiques (âge, sexe), le type de CE, le délai présumé entre l'ingestion du CE et la réalisation de l'extraction, le moyen utilisé pour l'extraction, la durée de la procédure, les complications liées à la procédure et le succès ou l'échec pour chaque procédure.

## Résultats

**Figure 1:**
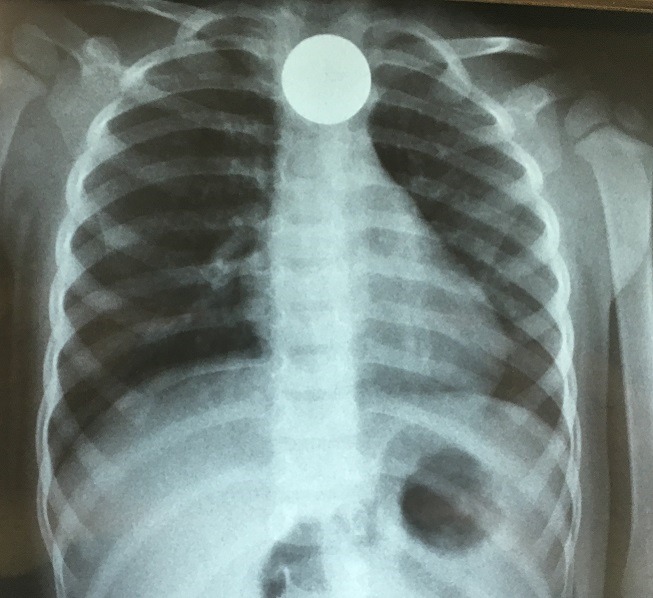
Radiographie pulmonaire de face montrant une pièce de 25 francs CFA bloquée dans le tiers supérieur de l’œsophage

37 patients ayant bénéficié d'une extraction de CE du tiers supérieur de l'œsophage étaient inclus. Tous les patients présentaient une dysphagie aux solides, justifiant l'extraction du CE. Le CE était identifié dans 100% des cas grâce à la réalisation d'une radiographie pulmonaire de face. Il s'agissait dans la majorité des cas d'une pièce de monnaie (Figure 1) et dans une moindre mesure de piles plates type pile « bouton » (Tableau 1), tous radio-opaques. L'âge médian était de 3 ans et l'on retrouvait une prédominance masculine (Tableau 2). Le délai médian entre le syndrome de pénétration présumé et la manœuvre d'extraction était de 3 jours (Tableau 2). Tous les patients bénéficiaient d'une anesthésie générale, 23 patients d'une anesthésie générale en ventilation spontanée (62,2%) et 14 d'une anesthésie générale avec intubation oro-trachéale (37,8%). Pour l'extraction, deux moyens étaient utilisés: pour 16 patients (43,2%) une sonde de Foley de calibre de 12 à 16 Fr munie d'un ballonnet de 25 ml était introduite sous laryngoscopie directe dans l'œsophage par la bouche. La sonde était ensuite poussée en distalité du CE à l'aveugle. Le ballonnet était alors gonflé avec de l'eau et la sonde était retirée délicatement jusque dans la cavité buccale. Le CE était ensuite extrait soit manuellement, soit à l'aide d'une pince de Magyll. (groupe “foley”); pour 21 patients (56,8%) une pince à calcul était directement introduite dans la bouche œsophagienne sous laryngoscopie directe jusqu'à la préhension du CE. La scopie était utilisée en temps direct pour aider à la préhension du CE pour 5 d'entre eux (23,8%). (groupe “pince à calcul”) Comparativement, il n'y avait pas de différence statistiquement significative entre les deux groupes en termes de nombre de tentatives moyen pour extraire le CE ni de durée médiane de procédure (Tableau 3). En revanche, 4 complications étaient notées après l'extraction à l'aide de la pince à calcul (3 bronchospasmes et 1 saignement local de la bouche œsophagienne ayant nécessité plusieurs aérosols d'adrénaline avant son tarissement). Deux échecs étaient relevés dans chaque groupe. Dans le groupe “foley”, il était dans l'un des cas nécessaire de changer de procédure en utilisant une pince à calcul avec succès et dans l'autre il était réalisé une cervicotomie pour l'extraction du CE. Inversement, dans le groupe “pince à calcul”, pour 'un des patients l'utilisation de la sonde de foley pour l'extraction était également un échec et c'était finalement l'utilisation non conventionnelle d'un cystoscope rigide et d'une pince qui permettait d'extraire le CE. Dans l'autre cas une cervicotomie était également réalisée après échec de la pince à calcul et de la sonde de Foley.

**Tableau 1:** Type de corps étranger retrouvé

Pile	3 (8,1%)
Pièce	34 (91,9%)

**Tableau 2:** Données démographique s et délai entre le syndrome de pénétration et l’extraction

n	sexe H/F	âge (années) (médiane [min-max])	durée enclavement (jours) (médiane [min-max])
37	20/17	3 [0,5-15]	3 [0-90]

**Tableau 3:** Tableau comparatif des deux types d’extractions réalisées

	Foley	pince	p
**n**	16 (43,2%)	21 (56,8%)	
nombre de tentatives (moyenne [min-max])	1,75 [1-3]	1,42 [1-4]	0,12
Durée (min) (médiane [min-max])	15 [5-90]	15 [2-120]	0,93
complications	0	4 (19%)	0,12
Echec	2 (12,5%)	2 (9,5%)	1

## Discussion

Dans notre population, les données épidémiologiques étaient similaires aux données de la littérature. Chez l'enfant, l'âge moyen est en effet de 2,6 ans, expliqué à la fois par le développement psychomoteur avec l'exploration de son environnement et par un début de la diversification alimentaire dès l'âge de 6 mois [3]. Comme dans la littérature, le sexe ratio retrouve une légère prédominance masculine [4]. Si la mortalité des CE ingérés est actuellement inférieure à 1% dans les pays industrialisés, les données épidémiologiques concernant les pays Africains sont pauvres [1]. La morbi-mortalité des accidents domestiques dans la population pédiatrique dans les pays en développement est sous-estimée et reste un problème de santé publique majeur [5,6]. Concernant l'ingestion de CE, les complications peuvent être liées soit à la nature des corps étrangers (objets contondants avec risque de perforation, ingestion de pile au lithium avec risque de libération de substance caustiques ou de brûlure électromécanique au contact) [7, 8], soit liées à la procédure d'extraction [9]. A court terme, les complications que l'on peut rencontrer dans ce contexte sont la perforation de l'œsophage, qui bien que rare est de pronostic sombre [10]. Une autre complication est l'impossibilité de retirer le CE, après une ou plusieurs tentatives, d'autant plus s'il est enclavé depuis plusieurs jours ou plusieurs semaines. Il faudra alors savoir recourir à un traitement chirurgical, ce qui était le cas pour deux des patients de notre série. A moyen et long termes, les risques rencontrés sont plutôt rares (moins de 1%): la sténose de l'œsophage (soit directement par le corps étranger, soit secondaire à une sténose cicatricielle après extraction), la migration dans les tissus ou organes de voisinage [11,12] et la fistule responsable le plus souvent de médiastinite et de façon plus anecdotique de fistule œso-trachéale (surtout en cas d'ingestion de pile au lithium) [13] ou œso-aortique [14, 15]. Aucune de ces complications tardives n'a été retrouvée dans notre étude. Il s'agissait de complications mineures dans les 4 cas, directement imputables au geste d'extraction et à l'anesthésie.

Même si les CE ingérés sont évacués spontanément dans 80 à 90% des cas, ils nécessitent des manœuvres d'extraction non chirurgicales dans 10 à 20% et pour moins de 1% le recours à la chirurgie [2]. Si le recours à l'endoscopie est recommandé pour la prise en charge des CE du tiers supérieur de l'œsophage situés sous le muscle crico-pharyngien, d'autres alternatives étaient utilisées dans la population étudiée, en raison de l'absence de matériel d'endoscopie (seul un cystoscope rigide était disponible, sans matériel d'extraction adapté) et de l'absence de personnel formé à son utilisation. La sonde de Foley ou sonde à ballonnet sous contrôle radioscopique a été décrite pour la première fois en 1966, avec un taux de succès de 98% [1], mais elle ne peut s'appliquer qu'aux corps étrangers mousses. Son utilisation présente l'avantage d'être moins risquée, significativement moins coûteuse et plus rapide que l´endoscopie. Dans notre étude, le taux d'échec était de 12,5%. L'utilisation systématique de la radioscopie pourrait augmenter les chances de succès. La pince à calcul était utilisée avec un taux d'échec de 9,5% dans la population étudiée. Cette technique ne peut néanmoins pas s'appliquer aux CE qui sont situés trop en distalité de l'œsophage. Elle nécessite un opérateur entraîné et l'anesthésiste-réanimateur, habitué à la gestion des voies aériennes supérieures est un opérateur privilégié pour cette technique. Le principal risque de cette technique est de léser et de perforer l'œsophage avec la pince. L'utilisation systématique de la scopie pourrait également faciliter la procédure et limiter le risque d'échec. D'autres techniques qui n'étaient pas utilisées dans notre étude sont décrites dans la littérature. C'est le cas du cathéter à extrémité aimantée, qui peut être indiqué pour l'extraction des CE ferreux [16]. Ce dernier est descendu sous contrôle radioscopique au contact du CE. L'avantage est son extrême simplicité, mais le risque de passage dans les voies aériennes supérieures nécessite de façon systématique une anesthésie avec intubation [1]. Certains auteurs préconisent un traitement médical par glucagon [1], permettant ainsi le relâchement du sphincter inférieur de l'œsophage. Il n'est néanmoins pas indiqué dans la prise en charge des CE du tiers supérieur de l'œsophage. Enfin, la chirurgie est indiquée devant une complication aiguë ou devant l'échec de l'extraction instrumentale. Les données de la littérature retrouvent un taux de recours à la chirurgie de seulement 1% (99% des CE œsophagiens sont extraits par endoscopie), alors que dans notre population ce taux est de 5,4%. Ceci peut s'expliquer par l'absence de matériel d'endoscopie et la sous-utilisation de l'appareil de radioscopie.

## Conclusion

L'extraction de CE du tiers supérieur représente un réel défi pour le médecin ne disposant pas de matériel d'endoscopie. Si l'évacuation des CE se fait le plus souvent spontanément, nombreux sont les cas nécessitant une extraction instrumentale. Les indications doivent être bien connues afin de ne pas aggraver la symptomatologie et s'exposer au risque d'iatrogénie. En l'absence de matériel d'endoscopie et de médecin gastro-entérologue, l'utilisation de techniques alternatives fiables est de mise. C'est le cas de la sonde à ballonnet et de l'utilisation de la pince à calcul. Ces deux techniques sont réalisées sous anesthésie générale afin de limiter le risque d'inhalation. L'utilisation simultanée de la radioscopie pour ces deux procédures pourrait limiter le risque d'échec et le recours à la chirurgie.

### Etat des connaissances actuelle sur le sujet

- L'ingestion de CE n'est pas une pathologie rare;
- L'évacuation est le plus souvent spontanée;
- En cas d'indication d'extraction instrumentale, le recours à l'endoscopie est de mise. D'autres moyens existent en cas d'indisponibilité de l'endoscopie.

### Contribution de notre étude à la connaissance

- L'utilisation de la sonde de Foley et de la pince à calcul pour l'extraction des CE du tiers supérieur de l'œsophage sont des méthodes fiables, utilisables par des médecins anesthésistes-réanimateurs;
- L'utilisation systématique de la radioscopie pourrait limiter le risque d'échec;
- La présence d'un chirurgien viscéraliste est nécessaire en cas d'échec ou de complication liée à la procédure.

## Conflits d’intérêts

Les auteurs ne déclarent aucun conflit d'intérêt.
